# Non-invasive hemodynamic evaluation by Doppler echocardiography

**DOI:** 10.5935/0103-507X.20180055

**Published:** 2018

**Authors:** António Gaspar, Pedro Azevedo, Roberto Roncon-Albuquerque Jr

**Affiliations:** 1 Serviço de Cardiologia, Hospital de Braga - Braga, Portugal.; 2 Serviço de Medicina Intensiva, Centro Hospitalar São João, EPE - Porto, Portugal.

**Keywords:** Echocardiography, Doppler, Hemodynamic

## Abstract

The approach for treating a hemodynamically unstable patient remains a diagnostic and therapeutic challenge. Stabilization of the patient should be rapid and effective, but there is not much room for error. This narrow window of intervention makes it necessary to use rapid and accurate hemodynamic evaluation methods. Echocardiography is the method of choice for the bedside evaluation of patients in circulatory shock. In fact, it was intensive care physicians who recognized the potential of Doppler echocardiography for the initial approach to patients in circulatory failure. An echocardiogram allows rapid anatomical and functional cardiac evaluation, which may include non-invasive hemodynamic evaluation using a Doppler study. Such an integrated study may provide data of extreme importance for understanding the mechanisms underlying the hemodynamic instability of the patient to allow the rapid institution of appropriate therapeutic measures. In the present article, we describe the most relevant echocardiographic findings using a practical approach for critical patients with hemodynamic instability.

## INTRODUCTION

A hemodynamically unstable patient is a critically ill patient with the potential to progress to circulatory shock and death within hours. Thus, rapid, accurate and reproducible diagnostic methods should be used to institute appropriate therapeutic measures.

Through anatomic and functional cardiac evaluation, an echocardiogram can provide data of extreme importance for understanding the mechanisms underlying the hemodynamic instability of a patient or even in the context of cardiorespiratory arrest, allowing appropriate therapeutic measures to be rapidly established.^(1,2)^

It was intensive care physicians who recognized the potential of Doppler echocardiography (echo-Doppler) for the initial approach to patients in circulatory failure.^(3)^ Hemodynamic evaluation with Doppler study was added to the classically performed anatomical and functional evaluation, which included cardiac output (CO), central venous pressure (CVP) estimation, peripheral vascular resistance (PVR) calculation, and left ventricle (LV) filling pressure estimation. Over the last few decades, Doppler echocardiography has become an increasingly widespread tool to the intensive care of patients in circulatory shock.^(1-5)^

**Figure 1:**
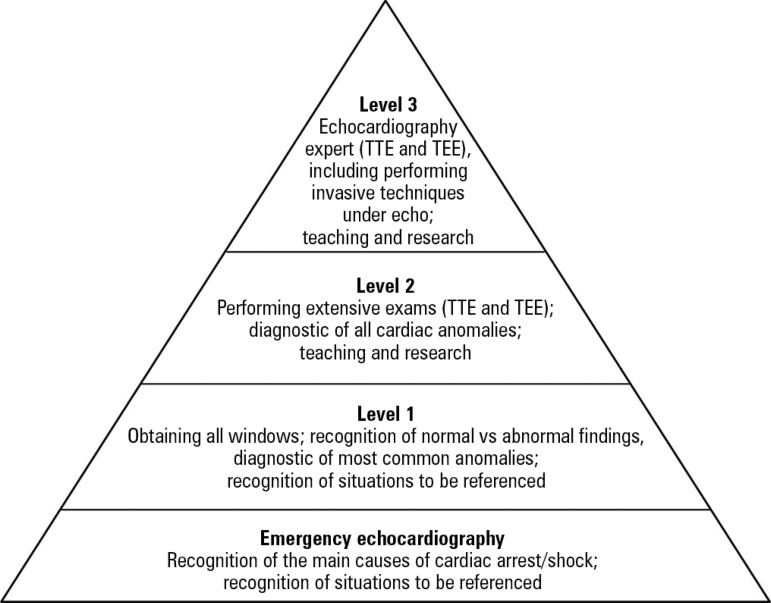
Levels of competence in echocardiography. TTE - transthoracic echocardiography; TEE - transesophageal echocardiography. Source: Adapted from Price S, Via G, Sloth E, Guarracino F, Breitkreutz R, Catena E, Talmor D; World Interactive Network Focused on Critical UltraSound ECHO-ICU Group. Echocardiography practice, training and accreditation in the intensive care: document for the World Interactive Network Focused on Critical Ultrasound (WINFOCUS). Cardiovasc Ultrasound. 2008;6:49.^(6)^

The competencies required to perform an echocardiogram are usually separated into three levels of experience, and a prior, more basic level of emergency echocardiography is recognized.^(6)^ The latter refers us to the visual identification of specific findings, allowing for specific diagnoses, such as cardiac tamponade or pulmonary embolism. Emergency echocardiography can be considered a basic competency of an emergency physician that is integrated into the initial assessment protocols necessary for severe patients, especially in the context of advanced life support. However, more thorough examinations require higher levels of competence. Figure 1 summarizes the levels of competence of echocardiography.

This review is primarily intended for physicians performing emergency and level I echocardiography.

### Circulatory shock: a common presentation for diverse hemodynamic entities

Circulatory shock is the clinical expression of a state of circulatory failure resulting in inadequate tissue perfusion and poor cellular oxygen supply, which can lead to injury and cell death.^(7)^ It is classically characterized by persistent hypotension (systolic blood pressure < 90mmHg) and peripheral hypoperfusion (altered state of consciousness, cold and cyanotic extremities and/or decreased urine output of < 0.5mL/kg/hour).^(7,8)^ In this context, hyperlactacidemia is a typical analytical finding resulting from abnormal cellular oxygen metabolism.

There are several causes of shock with different hemodynamic profiles and, consequently, different therapeutic needs. Some hemodynamic profiles are classically associated with some etiologies, such as cardiogenic shock (low CO and increased PVR in the presence of elevated ventricular filling pressures) and septic shock (increased CO and decreased PVR). However, the hemodynamic profiles of each type of shock are not always linear (Table 1).

**Table 1:** Hemodynamic characteristics according to the etiology of shock

Shock	RAT/CVP	PCWP	CO	PVR	EF
Hypovolemic	⇓	⇓	⇓	⇑	N/⇑
Cardiogenic	⇑	⇑	⇓	⇑	⇓
Vasoplegic	⇓	⇓	⇑	⇓	N/⇑
Septic	⇓	⇓	⇑/⇓	⇓	N/⇑/⇓
Neurogenic	⇓	⇓	⇓	⇓	⇓

RAT - right auricle pressure; CVP - central venous pressure; PCWP - pulmonary capillary wedge pressure; CO - cardiac output; PVR - peripheral vascular resistance; EF - ejection fraction; N - normal.

Cardiogenic shock has a component of systemic inflammation that can lead to a decrease in PVR, and patients with cardiogenic shock are more susceptible to septic complications.^(8-10)^ It should be noted that, in the SHOCK study, sepsis was suspected in 18% of patients, 74% of which developed positive blood cultures. These patients had a lower PVR days before the diagnosis of infection.

However, septic shock may present as a hypodynamic state, as characterized by a transient decrease of the ejection fraction (EF) and lower-than-expected values for CO and PVR, in approximately a third of cases.^(3,11,12)^ Thus, besides the etiology of shock, it is necessary to determine the precise hemodynamic profile of each patient, which is not limited to the observed hypotension.

Adequate and early support of patients who are in shock is essential to prevent multiorgan dysfunction.^(7)^ However, it is also true that exaggerated or misguided resuscitation, for example through excessive fluid therapy, can lead to deleterious effects.^(13)^

Diagnostic evaluation should be as accurate and objective as possible to quickly determine the supportive measures most appropriate to the etiology of the shock and its hemodynamic profile. Doppler ultrasound has been used as the diagnostic method of choice in this context.

### Practical approach for the assessment of hemodynamically unstable patients via Doppler echocardiography

The clinical context and the physical examination often make it possible to infer the most probable etiology of hemodynamic instability for a patient. In these patients, echo-Doppler is an important tool for the stratification of shock and the definition of the most adequate supportive therapy. In other patients, initially, circulatory failure has an unexplained cause. In the latter context, echo-Doppler is a very attractive diagnostic method for the initial study of the patient.

In the present article, we describe the most relevant echocardiographic findings using a practical approach directed at patients with hemodynamic instability.

### Cardiac chambers and valvular devices

**Figure 2:**
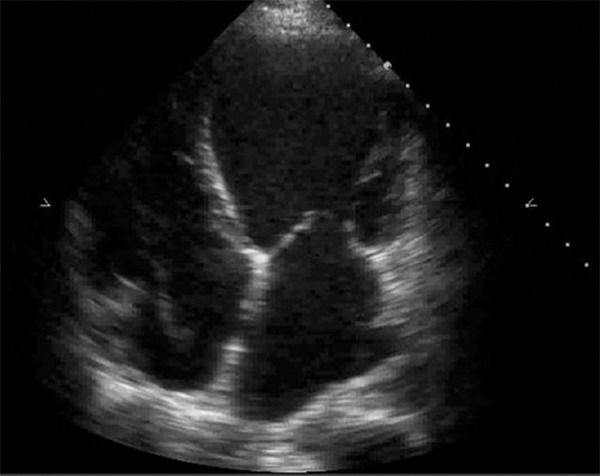
Dilated cardiomyopathy.

The evaluation of the morphology of the cardiac chambers allows us to infer the time of evolution of an eventual cardiopathy. The presence of the dilatation of these chambers favors a chronic pathology, while cardiac chambers of normal size point to an acute or subacute pathology (Figure 2).

Moreover, the anatomical and functional evaluation of valves can reveal a regurgitant or stenotic lesion significant enough to justify or at least contribute to the hemodynamic instability of the patient. A description of the qualitative and quantitative methods for this evaluation can be found in various recommendations of the European Association of Echocardiography.^(14-16)^

It is important to remember that acute and subacute valvular pathologies are the most frequent causes of significant hemodynamic instability due to the lack of time for the physiological mechanisms of adaptation to act. Typically, important valvular dysfunction is observed with non-dilated cardiac chambers.

**Figure 3:**
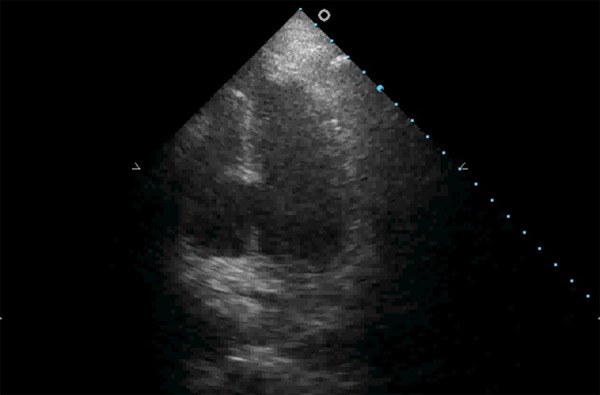
Interventricular septum rupture under acute inferior wall myocardial infarction.

When hemodynamic instability occurs under acute myocardial infarction (AMI), particular emphasis should be given to the observation of mechanical complications, namely the rupture of the free wall, interventricular septum or papillary muscle (Figure 3).

### Left and right ventricular systolic function

Evaluation of the systolic function of both ventricles is usually part of the assessment of any hemodynamically unstable patient. In urgent cases, there are several methods available for assessment of LV systolic function, including ejection fraction (EF) by Simpson's method, mitral annular plane systolic excursion (MAPSE), and fractional shortening and velocity of the S wave in the mitral annular plane by tissue Doppler (TDI: tissue Doppler imaging).^(17,18)^

The systolic function of the right ventricle (VD) can be assessed by systolic excursion of the tricuspid annular plane (TAPSE: tricuspid annular plane systolic excursion), fraction of the shortening area (FAC: fractional area change) and velocity of the S wave in the tricuspid annular plane by TDI.^(19)^

The evaluation of systolic function also includes the study of segmental kinetics. Changes in LV regional motility often show the presence of coronary disease, which may or may not be the cause of the instability under study. Hypokinesis of the medial-basal segments of the RV free wall, known as McConnell's signal, is a characteristic finding of acute pulmonary thromboembolism (PTE) and RV infarction. Its presence increases the specificity of other echocardiographic findings for the diagnosis of PTE, namely dilation of right heart chambers and pulmonary hypertension.^(20)^

### Pericardial effusion

Echocardiogram is the method of choice for diagnosing and evaluating pericardial effusion.

 According to its thickness at diastole, circumferential effusion may be generically classified as being very small (< 5mm), small (5 to 10mm), moderate (10 to 20mm) or large (> 20mm) in volume. Sometimes, especially after cardiac surgery, effusion may accumulate preferentially over one or two cardiac chambers.

**Figure 4:**
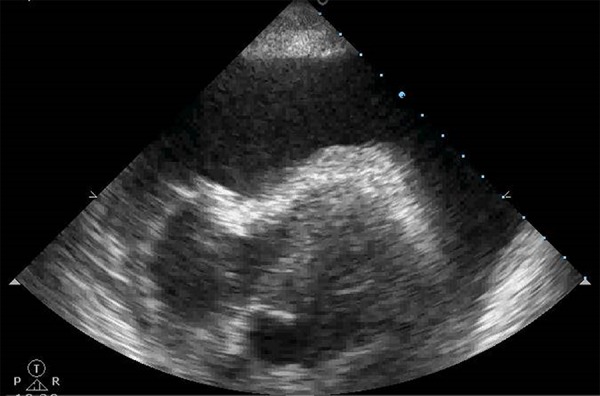
Cardiac tamponade.

The accumulation of fluid in the pericardial space leads to an increase in intrapericardial pressure, which, in turn, may limit cardiac filling if the intracardiac filling pressure is exceeded, leading to cardiac tamponade. Cardiac tamponade is a hemodynamic continuum whose maximum expression is circulatory failure (Figure 4).

Although cardiac tamponade is a clinical diagnosis, the latter should be confirmed via echocardiography by demonstrating pericardial effusion with evidence of repercussion in cardiac function, namely, in right ventricular filling: dilation of the inferior vena cava (IVC) and suprahepatic veins and the absence of their respiratory variability; exaggerated respiratory variability (> 30%) of the transtricuspid and transmitral flows; and finally, diastolic collapse of the right chambers (initially the auricle and, at a later stage, the ventricle).^(21)^

### Determination of blood volume and response to fluid therapy

Classically, fluid therapy is the first therapeutic line in the initial phase of approaching a hemodynamically unstable patient in sepsis and septic shock.^(22)^ The purpose of fluid therapy is preload optimization, one of the four major determinants of cardiac function (the other three are post-load, contractility and heart rate). However, excessive fluid therapy in a patient whose preload is already high can have deleterious effects, especially for the right heart.^(13,23)^

Several echocardiographic parameters have been used to estimate blood volume. Obliteration in LV systole may be an extreme sign of hypovolemia (kissing papillary muscles sign). However, if this is not found, other echocardiographic data should be collected and adjusted to the clinical context of the patient to guide the resuscitation measures. Moreover, the presence of dilated chambers does not exclude eventual response to fluid therapy.

The diameter of the IVC and the analysis of its respiratory variability have been used to infer the pressure of the right auricle and, consequently, the CVP.^(24)^ However, estimation of blood volume using this method has several limitations, especially in patients with previous respiratory disease or under positive pressure ventilation. Generally, dilated IVC without respiratory variability suggests elevated CVP, whereas reduced-diameter IVC with great variability suggests reduced CVP.

However, more important than the estimate of blood volume is the determination of if its increase will bring an increase in CO.

In spontaneously breathing patients, an IVC distensibility index ([maximum diameter - minimum diameter]/maximum diameter) > 40% was associated with fluid therapy response, and a value < 40% did not exclude the response.^(24)^ Additionally, in patients breathing spontaneously, the increase in CO by 15% with passive raising of the lower limbs above 30º (passive leg raising) was a predictor of response to volume, with 77% sensitivity and 100% specificity.^(25)^ In patients under mechanical ventilation, an IVC distensibility index ([maximum diameter - minimum diameter]/[maximum diameter + minimum diameter)/2]) > 12% allowed responders to volume expansion to be identified with positive and negative predictive values of 93% and 92%, respectively.^(26)^ However, this acuity in predicting the response to fluid therapy was not observed in other studies with more heterogeneous populations, which points to the limitations of the generalization of these indices.^(27)^

The evaluation of the response to fluid therapy can also be performed by evaluating the ejection volume (VolEj) via echocardiography before and after fluid challenge administration. The increase in VolEj by > 15% in response to a fluid challenge probably provides the most evidence of the response to fluid therapy.^(28)^ By considering that CO depends on the variations not only of VolEj but also of CF, we advise verifying an increase of CO by > 15% as a criterion of response to fluid challenge. The calculations of VolEj and CO by echocardiography, as well as ways of streamlining them in successive re-evaluations, are explained in detail in the following section.

The role of echocardiography in the orientation of fluid therapy can still be complemented by pulmonary ultrasonography.^(29)^ The appearance of pattern B on lung ultrasound may allow the fluid therapy to be stopped before it becomes excessive and deleterious. Based on this premise, Lichtenstein developed the Fluid Administration Limited by Lung Sonography (FALLS) protocol. While the purpose of this review is not to address pulmonary ultrasonography, we recommend reading Lichtenstein's recent review of the subject.^(29)^

Many of the parameters used in determining blood volume and fluid therapy orientation are not specific and, as such, have limitations.^(27,28)^ Various clinical findings, physical examination and ultrasound should be integrated to increase the diagnostic acuity and predictive value of various parameters.

### Determination of cardiac output and peripheral vascular resistance

Although the evaluation of biventricular systolic function is important for the evaluation of patients in shock, it has some limitations: there is no linear correlation between biventricular systolic function and the hemodynamic state, as there may be a severe depression of LV systolic function without circulatory shock and, conversely, there may be circulatory shock with only moderate depression of LV systolic function.^(8)^ CO is undoubtedly the most important hemodynamic parameter for critical patients.

**Figure 5:**
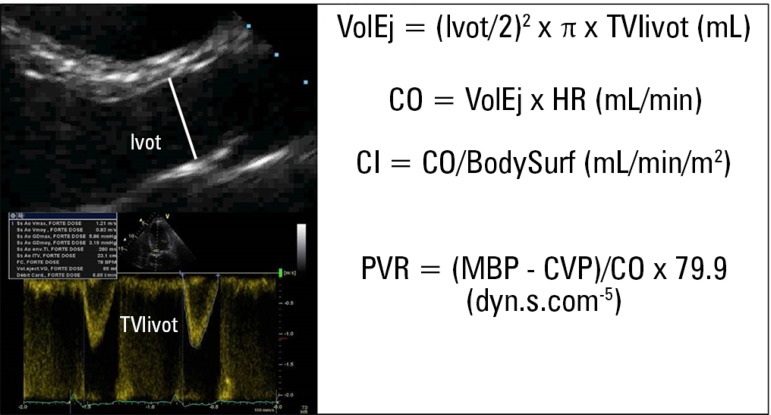
Calculation of cardiac output and peripheral vascular resistance via Doppler echocardiography. LVOT - left ventricular outflow tract in parasternal long axis; TVIlvot - time-velocity integral of the flow of the left ventricle outflow tract in the parasternal long axis in the apical 5-chamber; VolEj - ejection volume; CO - cardiac output; HR - heart rate; CI - cardiac index; BodySurf - body surface; PVR - peripheral vascular resistance; MBP - mean blood pressure.

Echo-Doppler allows us to calculate CO relatively easily (Figure 5). The area of the cross-sectional cut of the LV outflow tract (lvot), which is a circle, can be calculated using the formula (Dlvot/2)^2^ × π, where Dlvot is the diameter of lvot measured along the parasternal long axis. The area of ​​lvot is multiplied by the time-velocity integral of lvot flow (TVIlvot), as measured at apical incidence 5 chambers at the same location as the lvot measurement, which gives us VolEj. In turn, CO is obtained by multiplying VolEj by HR. The cardiac index (CI) can be derived by dividing CO by body surface area (BodySurf). Thus, we have:

$$\begin{array}{c} \mathrm{VolEj}={\left(\mathrm{lvot}/2\right)}^{2}\times \pi \times \mathrm{TVI}\mathrm{lvot}\;(\mathrm{mL})\\ \mathrm{CO}=\mathrm{VolEj}\times \mathrm{HR}\;(\mathrm{mL}/\mathrm{minute})\\ CI=CO/\mathrm{BodySurf}\;(\mathrm{mL}/\mathrm{minute}/m^{2}) \end{array}$$

Cardiac output calculation via echo-Doppler was validated in several studies, where the thermodilution method was used as the gold standard.^(30,31)^ However, there are some potential sources of error in the estimation of CO, namely the measurement of lvot and the presence of arrhythmias causing great variability in TVIlvot. Thus, estimating CO via echo-Doppler should be seen more as as a semi-quantitative method, categorizing CO as very low, low, normal or high.

In turn, the CO estimate allows PVR to be calculated as follows:

$$\mathrm{PVR}=\left(\mathrm{MBP}-\mathrm{CVP}\right)/\mathrm{CO}\times 79.9\;\mathrm{dyn}.s.{\mathrm{cm}}^{-5}$$

The estimation of CO and PVR allows the hemodynamic profile of a given patient to be defined. Because blood pressure is derived from the product of CO and PVR, defining the hemodynamic profile (CO and PVR) allows the origin of hypotension to be defined, i.e., whether it is dependent on low CO and/or vasoplegia.

Echo-Doppler allows us to reassess the patient and assess the efficacy of therapeutics, mainly in the absence of continuous hemodynamic monitoring. For this purpose, it is suggested that the same lvot measurement is maintained, which essentially values the variations in the product TVI × HR as equivalent to CO. In fact, in serial evaluations, the variations in TVI × HR is what will be important in clinical practice.

### Pulmonary artery systolic pressure

Pulmonary artery systolic pressure (PASP) can be determined from tricuspid insufficiency. Using the modified Bernoulli equation (4 × velocity^2^), the pressure gradient between RV and the right auricle is obtained, which, together with right ventricular pressure, allows PASP to be estimated (in the absence of pulmonary stenosis or other obstruction of the RV outflow tract).

A PASP > 35mmHg suggests the presence of pulmonary hypertension, while values above 60mmHg suggest severe pulmonary hypertension.

**Figure 6:**
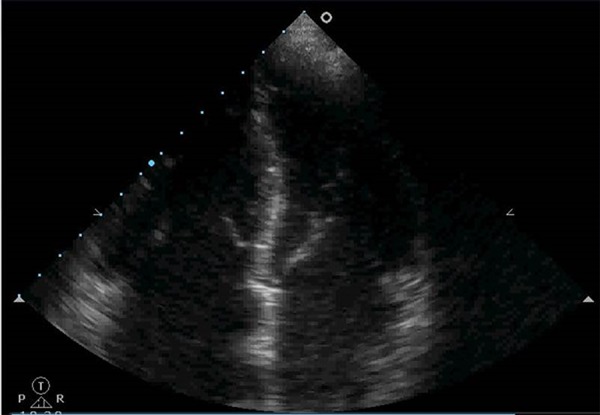
Right ventricular dilation with bulging of the interventricular septum towards the left ventricle under acute pulmonary thromboembolism.

The RV is a chamber designed to eject blood in a low pressure system and is intolerant of abrupt post-load increases.^(32,33)^ In emergency or intensive care settings, the two most frequent causes of acute right ventricular failure are PTE and acute respiratory distress syndrome (ARDS). In these cases, a normal RV cannot maintain normal function under abrupt rises in post-load. There is a 25% decrease in RV VolEj when the pulmonary artery mean pressure reaches approximately 30mmHg (corresponding to a PASP of approximately 45mmHg) and a marked and rapid decline in RV VolEj under, even slight, additional elevation of the afterload.^(33,34)^ The immediate compensatory response will be RV dilation, with bulging of the interventricular septum towards the LV to maintain the CO (Figure 6).

However, PASP determination is limited in certain situations, such as RV failure or the presence of free tricuspid insufficiency.^(35)^

### Estimation of left ventricular filling pressure

Left ventricle filling pressure (LVFP) is classically inferred by pressures obtained from the placement of a pulmonary catheter, mainly by means of pulmonary capillary wedge pressure (PCWP).

Echo-Doppler allows semiquantitative estimation of LVFP in normal or elevated conditions, and this approach was summarized in joint recommendations by the American Society of Echocardiography and European Association of Cardiovascular Imaging.^(36)^

**Figure 7:**
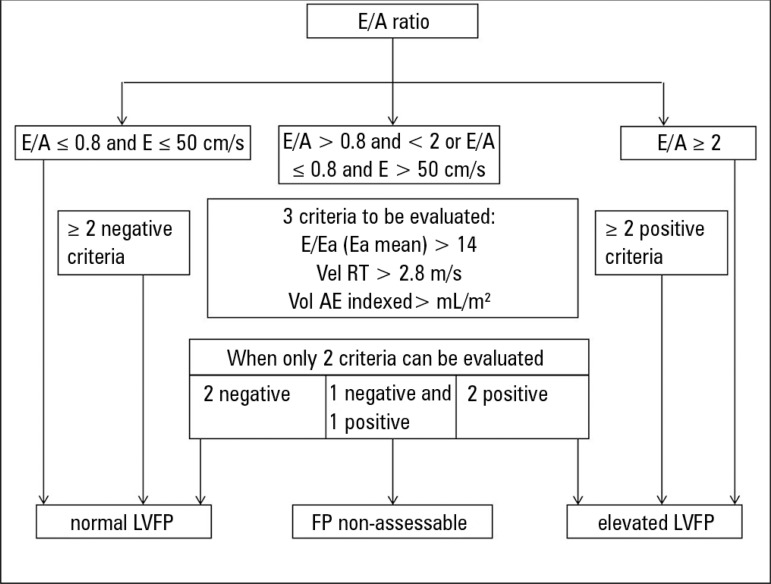
Estimation of left ventricular filling pressures. E/A - ratio between early filling velocity E of transmitral flow and late filling velocity A in pulsed Doppler; E/Ea - ratio between the early filling velocity E of the mitral flow in the pulsed Doppler and the early diastolic velocity Ea in the tissue Doppler collected at the level of the mitral annulus; Vel TR - velocity of tricuspid regurgitation; Vol LA - left auricle volume; LVFP - left ventricular filling pressures. Source: Adapted from Nagueh SF, Smiseth OA, Appleton CP, Byrd BF 3rd, Dokainish H, Edvardsen T, et al. Recommendations for the Evaluation of Left Ventricular Diastolic Function by Echocardiography: An Update from the American Society of Echocardiography and the European Association of Cardiovascular Imaging. J Am Soc Echocardiogr. 2016;29(4):277-314.^(36)^

The first parameter to be evaluated is transmitral flow, which is the ratio between the early filling velocity E of the transmitral flow and the late filling velocity A of the pulsed Doppler (E/A ratio). An E/A ratio < 1 with E < 50cm/s indicates normal LVFP, while an E/A ratio > 2 with an E-wave deceleration time < 150ms favors high LVFP. For an E/A ratio between 1 and 2 or E/A < 1 with a velocity of E > 50cm/s, additional parameters (e.g., the ratio between the early filling velocity E of the mitral flow in the pulsed Doppler and the early diastolic velocity Ea in tissue Doppler collected at the level of the mitral annulus, E/Ea; velocity of tricuspid regurgitation, Vel TR; and indexed volume of the left auricle) are necessary to estimate LVFP, and at least two of three parameters are necessary to define LVFP (Figure 7).

There are, however, limitations for the estimation of LVFP, especially in patients with advanced heart failure and greatly increased ventricular volumes.^(37)^ In these cases, it may be necessary to complement the evaluation of the patient with invasive methods for the validation of the echocardiographic evaluation.

In the context of intensive care, the evaluation of diastolic function and the consequent estimation of LVFP can be simplified by considering only the early relaxation velocity Ea of tissue Doppler and the E/Ea ratio.^(38)^ Based on these two parameters, diastolic dysfunction can be assumed when Ea < 8cm/s and E/Ea > 14. Despite some limitations, this simplified assessment has been shown to play a role in the stratification of critically ill patients, such as patients with severe sepsis, for whom the presence of diastolic dysfunction has identified patients with poor prognosis.^(39)^ Diastolic dysfunction may also allow the identification of patients with a higher probability of failure at ventilatory weaning.^(40)^ Finally, evaluation of diastolic function may also play a role in the orientation of resuscitation with fluid therapy, especially in limiting it in septic patients.^(41)^

### Algorithm for initial assessment of hemodynamically unstable patients

**Figure 8:**
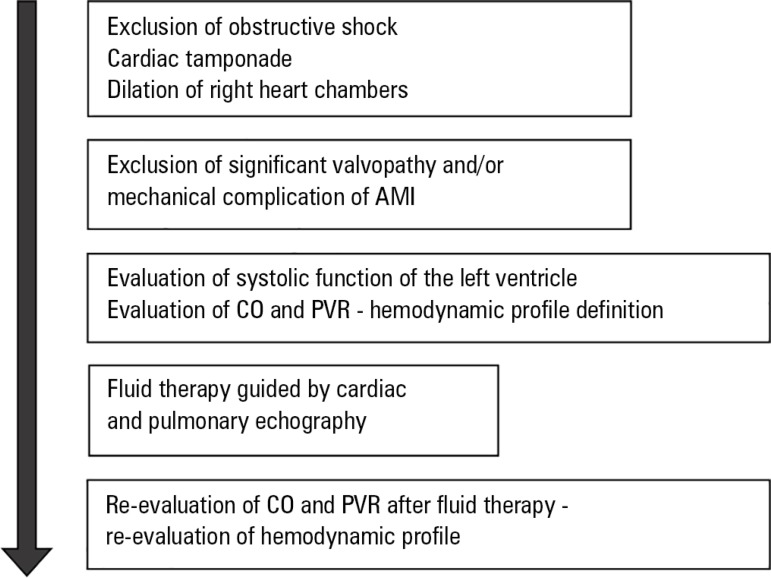
Algorithm for systematic echocardiographic evaluation of hemodynamically unstable patients. AMI - acute myocardial infarction; CO - cardiac output; PVR - peripheral vascular resistance. Source: Adapted from: Lichtenstein DA. BLUE-protocol and FALLS-protocol: two applications of lung ultrasound in the critically ill. Chest. 2015;147(6):1659-70.^(29)^

In figure 8, we propose an algorithm for the systematic evaluation of patients with hemodynamic instability.

Some findings of the echocardiographic examination, mainly cardiac tamponade, pulmonary embolism with acute *cor pulmonale* and severe left ventricular dysfunction, are easy to see and may be suited for emergency echocardiography. Other elements are more complex and require a higher level of training (level I at least), as is the case for CO calculation and LVFP estimation.

### Role of transesophageal echocardiogram

Transthoracic echocardiography (TTE) has good diagnostic accuracy in the evaluation of critically ill patients, even for patients under mechanical ventilation; therefore, it is the first option. However, it is not always possible to acquire adequate TTE images. Frequent causes of poor transthoracic echocardiography include obesity, emphysema, elevated levels of positive expiratory pressure or the presence of dressings and surgical drains. In this context, transesophageal echocardiography (TEE) is the ideal alternative.

Transesophageal echocardiography remains the reference echocardiographic examination in situations such as valvular prosthesis dysfunction, suspected infective endocarditis, and aortic dissection and in the presence of intracardiac *shunts* or cardioembolic sources. However, TEE requires a more advanced level of training in echocardiography (at least level I and, preferably, higher levels).^(6)^

## CONCLUSION

Echo-Doppler is the ideal tool for the initial approach to patients with hemodynamic instability. In addition to morphological and functional cardiac characterization, it allows a comprehensive evaluation of the hemodynamic status of the critically ill patient.

Performing echo-Doppler is often necessary in the evaluation of hemodynamically unstable patients. Echo-Doppler used as a hemodynamic tool allows a great deal of information to be obtained, which gives it not only a diagnostic role but also a therapeutic role, by helping define circulatory resuscitation strategies.

Finally, echo-Doppler should not be viewed as a substitute for continuous, invasive or semi-invasive monitoring methods. As complementary methods, echo-Doppler is naturally preferred in the initial evaluation of unstable patients, while continuous methods play a role in the continuous monitoring of patients.
